# Conventional Synthetic Disease-Modifying Anti-Rheumatic Drugs and the Risk of Vascular Dementia in Patients with Spondyloarthritis: A Database Cohort Study

**DOI:** 10.3390/jcm12030950

**Published:** 2023-01-26

**Authors:** Yu-Hao Lee, Shih-Wei Huang, Chih-Kuang Chen, Jia-Pei Hong, Yi-Wen Chen, Hui-Wen Lin

**Affiliations:** 1 Department of Physical Medicine and Rehabilitation, Shuang Ho Hospital, Taipei Medical University, New Taipei 23561, Taiwan; 2 Department of Physical Medicine and Rehabilitation, School of Medicine, College of Medicine, Taipei Medical University, Taipei 11031, Taiwan; 3 Graduate Institute of Sports Science, National Taiwan Sports University, Taoyuan City 33301, Taiwan; 4 Department of Physical Medicine and Rehabilitation, Chang Gung Memorial Hospital at Taoyuan, Taiwan School of Medicine, Chang Gung University, Taoyuan 333, Taiwan; 5 Department of Mathematics, Soochow University, Taipei 11102, Taiwan; 6 ICF Research Center, Shuang Ho Hospital, Taipei Medical University, New Taipei 23561, Taiwan

**Keywords:** axial spondyloarthritis, ankylosing spondylitis, dementia, DMARDs, population-based study

## Abstract

Axial spondyloarthritis (axSpA) is a chronic inflammatory rheumatic disease that mainly affects the axial bones, and dementia is characterized by a decline in cognitive function, leading to dependence in everyday activity. Although the association between dementia and ankylosing spondylitis has been investigated, the influence of axSpA medication on dementia risk is unclear. The aim of this study was to investigate the risk of dementia among axSpA patients and if the conventional synthetic disease-modifying anti-rheumatic drugs (csDMARDs) can reduce the risk of dementia. Patients with axSpA whose data were recorded during 2004–2008 and who were followed up until the end of 2010 were recruited. A control cohort was matched by age and sex. A Cox multivariate proportional hazards model was applied to analyze the risk factors for dementia. The hazard ratio (HR) and adjusted HR (aHR) were estimated between the study and control cohorts. The effects of csDMARDs and steroid use on the risk of different types of dementia were also analyzed. In total, 2341 and 11,705 patients constituted the axSpA and control cohort, respectively. The axSpA cohort had a greater risk of vascular dementia (aHR = 2.09 (1.36–3.20). The risk of dementia (aHR = 1.01 (0.55–1.85) did not significantly differ between patients with axSpA who received csDMARDs. In conclusion, patients with axSpA are at a risk of vascular dementia, which could be reduced by csDMARDs.

## 1. Introduction

Axial spondyloarthritis (axSpA) is a chronic inflammatory rheumatic disease that mainly affects axial bones, such as the spine or sacroiliac joints. Visual indications of structural lesions on images of the spine or the sacroiliac joints indicate a diagnosis of ankylosing spondylitis (AS) [1]. Reactive arthritis and psoriatic arthritis are the other types of spondyloarthritis (SpA) in addition to AS. The absence of structural lesions on radiographic images indicates a diagnosis of nonradiographic axSpA. SpA typically presents in the second to third decade of life, and patients with human leukocyte antigen B27 (HLA-B27) are usually diagnosed 5 years earlier than those without the HLA-B27 antigen [2,3]. Studies have reported a 0.20% prevalence of SpA (including peripheral forms) in Southeast Asia and a 1.61% prevalence in the northern Arctic [4]. Men are more likely to develop AS than women, with a ratio of approximately 2:1 to 3:1, respectively. However, no differences in gender distribution have been observed among patients with nonradiographic axSpA [3,5]. Patients with axSpA usually experience chronic back pain and stiffness in the pelvis and lower back. These symptoms of axSpA usually appear before the age of 40 years and may hamper an individual’s work and increase their medical expenses [1,6]. Previous studies have also discovered that patients with axSpA have a higher risk of developing mental disorders such as depression, anxiety, and sleep disturbances [7,8]. A recent study also reported that patients with AS had a higher prevalence of dementia [9].

Dementia is a common disease that affects older adults. It is characterized by a gradual decline in cognitive function involving one or more areas, eventually causing impaired function and dependence, accompanied by memory loss, personality changes, and disorientation [10]. It is a progressive neurodegenerative disease that affects not only patients but also their families, leading to a heavy burden on society and the healthcare system. According to the World Alzheimer’s Disease Report 2015, approximately 46 million individuals have dementia, and this number is expected to reach 131 million by 2050. The prevalence of developing dementia doubles every five years after the age of 65 years [11]. Dementia is a leading cause of disability among older adults, which hinders their everyday activity and social participation [12,13].

Although the association between dementia and AS was investigated in a previous study, the influence of medication for axSpA on the risk of subsequent dementia has not been studied [9]. This is important in order to prevent the development of dementia among patients with axSpA. We hypothesized that medications such as conventional synthetic disease-modifying anti-rheumatic drugs (csDMARDs) can lower the risk of dementia among patients with axSpA. Furthermore, glucocorticoids has an anti-inflammatory effect and the influence on risk of dementia among axSpA patients is not well investigated. Therefore, we conducted this longitudinal population-based study to identify the risk of dementia and the influence of axSpA-related medications on this risk.

## 2. Materials and Methods

### 2.1. The Data Source

Data from the National Health Insurance Research Database (NHIRD) of Taiwan, which is maintained by the National Research Agency and is to be used only for research, were analyzed in this cohort study. The NHIRD contains the health insurance data of more than 99% of the population in Taiwan. It covers health insurance-related information regarding disease diagnoses, insurance claims of outpatient and inpatients, medication prescriptions, interventions, surgeries, and physical examinations. The data were deidentified, and the requirement for informed consent was waived owing to the retrospective longitudinal observational nature of the databased study; the study was approved by the institutional review board of a university in Taipei (N202103132). 

### 2.2. Axial SpA Study Group Selection and Exclusion Process

**Figure 1:**
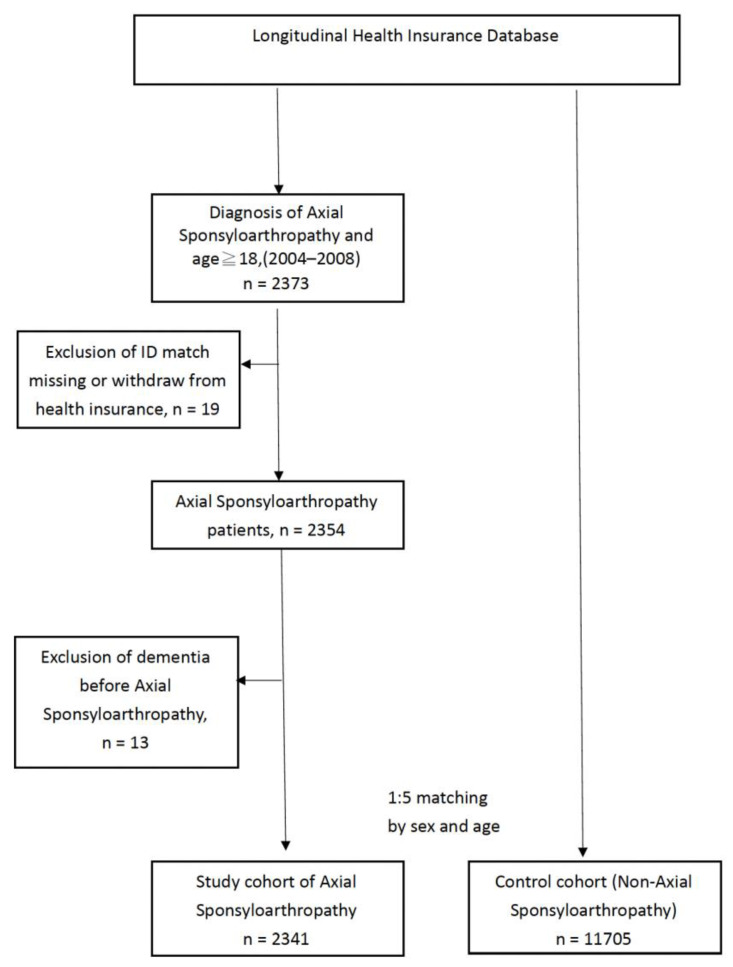
Study flow chart.

In NHIRD, diseases are recorded according to the diagnosis codes in the International Classification of Diseases, Ninth Revision (ICD-9). We collected the data of patients with axSpA aged more than 18 years with an ICD-9-Clinical Modification (CM) code. The diagnosis of axSpA was based on diagnostic code ICD-9-CM 720.0 during 1 January 2004 to 31 December 2007 from outpatient and inpatient claims data (*n* = 2373). In addition, only the data for at least two consecutive primary diagnosis codes for axSpA were collected. ICD codes 720.8 (other inflammatory spondylopathies) and 720.9 (unspecified inflammatory spondylopathy, site unspecified), which are not defined specific for ankylosing spondylitis, were not enrolled in the study cohort. Individuals diagnosed with dementia before the index date, those with missing data, and those not matched with age- and sex-matched controls were excluded. Follow-up started on the index date and ended on the date of diagnosis of dementia, death, or end of the study (31 December 2010; Figure 1).

### 2.3. Non-axSpA Control Cohort

From the same index date, patients without axSpA diagnostic coding in the database were then matched by performing propensity score matching as the control cohort at a ratio of 4:1 to study cohort to alleviate the potential confounding effects of sex, age, urbanization level (level 1: most capitalized; to level 5: least capitalized), monthly income (0, 1–20,000 TWD, 20,001–40,000 TWD, and ≥40,001 TWD), and selected comorbidities on the incidence of dementia.

### 2.4. Outcome and Relevant Comorbidities

The study outcome was a diagnosis of dementia (ICD-9-CM code 331.0 or code 290.0–290.4, 294.1, or 294.2) or Alzheimer’s disease (AD), with at least two outpatient visits or one hospital admission within 1 year. The endpoint of follow-up was determined as the occurrence of dementia from the index date. The comorbidities analyzed in this study were diabetes mellitus (ICD-9-CM codes 250 and 251), hypertension (ICD-9-CM codes 401–405), coronary heart disease (ICD-9-CM codes 410 and 412), Parkinson’s disease (ICD-9-CM code 332), hyperlipidemia (ICD-9-CM codes 272.0–272.4), autoimmune disease (rheumatoid arthritis (RA) and systemic lupus erythematosus (SLE); ICD-9-CM codes 714.0 and 710.0, respectively), and thyroid disorders (ICD-9-CM codes 240–246), gout (ICD-9-CM 274), and COPD (ICD-9-CM codes 491, 492, and 496). Having undergone medication therapy with conventional synthetic disease-modifying anti-rheumatic drugs (csDMARDs)—such as methotrexate, sulfasalazine, azathioprine, hydroxychloroquine, and leflunomide (due to limited anti-TNF-α treatment codes can be obtained The NHIRD during the period of 2004 to 2007, we did not contain the anti-TNF-α treatment as one of the csDMARDs in this study)—and oral glucocorticoids—such as prednisolone and methylprednisolone—for more than 28 days was also considered as a confounder in this study. 

### 2.5. Statistical Analysis

To compare the demographic categorial variables and comorbidities, Pearson’s chi-square test was used for analysis between the study and control cohorts. Continuous variables were compared between the study and control cohorts by independent *t* test. A Cox model with adjusted covariates (such as comorbidities, baseline variates, and medications such as corticosteroid and using csDMARD) was applied to analyze the risk of dementia (hazard ratio (HR) and adjusted hazard ratio (aHR)) between axSpA and non-axSpA cohorts. This model was further analyzed to determine the risk of vascular dementia and AD between the axSpA and non-axSpA cohorts. The use of medications, such as csDMARDs and oral glucocorticoids, was also analyzed under different models to determine the influence of medication use on the risk of development of different types of dementia among patients with axSpA. Kaplan–Meier hazard curves were drawn to present these analyses and the risk of dementia of the study cohort during the follow-up period. All data were analyzed in Stata (version 11; StataCorp, College Station, TX, USA) and SAS (version 9.1.3; SAS Institute, Cary, NC, USA) software. A *p* value < 0.05 was considered statistically significant.

## 3. Results

The control group was matched by age and sex at a ratio of 1:5 with respect to the incidence of dementia. Individuals diagnosed with dementia before the index date (*n* = 13), those with missing data, and those not matched with age- and sex-matched controls (*n* = 19) were excluded. After the selection process, the axSpA cohort comprised 2341 patients and the non-axSpA comparison cohort consisted of 11,705 age- and sex-matched participants (Figure 1). The percentage of male to female participants was 65.6% and 34.4% in both of the cohorts. The comorbidities observed in the patients were hypertension, coronary heart disease, hyperlipidemia, autoimmune diseases (RA and SLE), thyroid diseases, and gout (Table 1). 

The incidence of dementia in the axSpA and control groups were 334 and 233 per 100,000 person-years, respectively. The crude relative risk of dementia for the axSpA group was 1.53 (95% CI 1.08–2.17) relative to the control cohort. When the demographic variables and comorbidities were adjusted for, the adjusted risk of dementia was 1.55 (95% CI 1.08–2.23; Table 2). 

**Figure 2:**
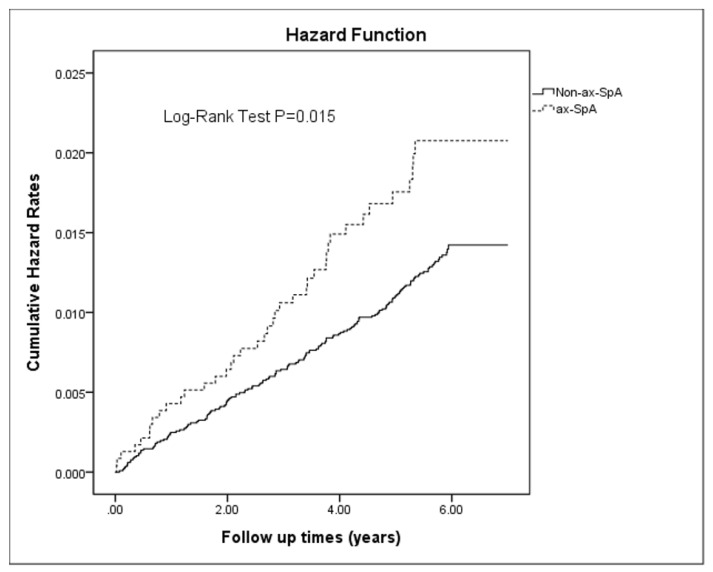
Kaplan–Meier curves for dementia risk between patients with and without axSpA at 7-year follow-up.

Kaplan–Meier hazard curves revealed that the cumulative incidence of dementia was higher in the axSpA group than in the comparison group (*p* < 0.05; Figure 2). 

Table 3 shows the results of Cox regression for patients with different types of dementia with or without csDMARDs. In patients with axSpA who did not receive csDMARDs, the adjusted HR was 1.98 (95% CI 1.32–2.99, *p* < 0.01). However, dementia risk did not differ between patients with axSpA who received csDMARDs and those without axSpA. The risk of developing AD did not differ between patients without axSpA and those with axSpA treated with or without csDMARDs. Patients with axSpA who were not receiving csDMARDs had a higher relative risk of developing vascular dementia (aHR, 2.09; 95% CI, 1.36–3.20, *p* < 0.01) relative to the control group. The relative risk of developing vascular did not differ between the control group and patients with axSpA who were receiving csDMARDs (Table 3). 

Patients with axSpA who were using glucocorticoids still had a higher risk of dementia; however, those receiving csDMARDs had a lower risk of dementia (Table 4).

## 4. Discussion

In our large-scale, longitudinal, and real-world data-based analysis, we observed that patients with axSpA have a greater-than-average risk of developing dementia. Upon further analysis, we found that patients with axSpA had a higher risk of developing vascular dementia; however, the risk of developing AD did not differ between patients with axSpA and their counterparts in the control cohort. The risk of developing vascular dementia did not differ between patients with axSpA who were receiving csDMARDs and their counterparts in the control cohort. However, patients with axSpA who were not receiving csDMARDs had a higher risk of developing vascular dementia than those receiving csDMARDs and those in the control cohort. In contrast with csDMARD use, which afforded a protective effect against dementia, glucocorticoids use was associated with a higher risk of developing dementia, regardless of the type. These findings regarding the effects of different medications on the risk of developing dementia among patients with axSpA can aid clinicians in dementia prevention. 

Although the mechanism underlying the development of dementia remains debated, some hypotheses and possible pathogeneses regarding the development of dementia among patients with axSpA have been suggested. Given that chronic inflammation is a characteristic finding among patients with axSpA, the inflammatory process may play a crucial role in elevating the risk of dementia. Previous epidemiological studies have reported an association between increased inflammatory biomarkers and dementia risk [14,15]. Moreover, chronic inflammation can induce atherosclerosis formation and lead to vascular dementia [16]. Previous studies have also indicated that proinflammatory cytokines such as interleukin (IL)-6, IL-1β, and tumor necrosis factor (TNF)- α can induce neurodegeneration of the brain [17,18,19]. Plaque formation, which is commonly observed in AD, can be initiated by amyloid β peptides, and previous studies on animals have reported that amyloid β peptide production can be increased by IL-1β and TNF-α [20]. The deposition of the extracellular amyloid is a well-defined pathogenesis and histological finding of AD [21]. Previous studies have also reported that patients with AS exhibited an increased serum amyloid level, which was a useful indicator of disease activity in these patients [22,23]. With the chronic pain and subsequent functional impairment caused by axSpA, patients are usually at a higher risk of developing psychiatric disorders, such as depression and anxiety [7]. Neuropsychiatric disorders are usually considered to be related to dementia, and depression is considered a risk factor for dementia [24].

A further analysis revealed that patients with axSpA had a risk of developing vascular dementia. In addition to chronic inflammation, amyloid deposition, and higher psychiatric disorders, reduced social participation and physical activities also contribute to the higher risk of dementia among patients with axSpA. These patients usually exhibit decreased physical activity from their early life and the progression of clinical symptoms with time [25]. Physical activity is associated with cognitive health [26], and decreased physical activity from a young age can increase the risk of developing dementia with time. Another hypothesis is that patients with axSpA have a higher risk for cerebrovascular accidents, which is a major etiology of vascular dementia [27]. 

Although our initial analysis revealed that patients with axSpA had a risk of developing dementia, regardless of type, a further analysis indicated that, first, these patients and those in the control cohort had a similar risk of developing AD and, second, this risk is not influenced by medication. Unlike another study, which reported that patients with AS had a higher-than-average risk of developing AD, our study did not find that patients with axSpA had a risk of developing AD [9]. The inconsistency in the findings between the previous study and our study could be attributable to the presence of different comorbidities in the patients, which were considered in our analysis. We also considered other autoimmune comorbid diseases, such as RA and SLE, which were considered as risk factors of dementia in previous studies [28]. As mentioned, amyloid is considered to be related to the disease activity of AS, and its deposition is considered a pathological finding of AD. We supposed that amyloid does not play a crucial role in the development of dementia among patients with axSpA. Moreover, csDMARD use did not influence AD risk among patients with axSpA, indicating that the chronic inflammation observed in axSpA is not associated with AD. 

We observed no difference in the risk of developing vascular dementia between patients with axSpA who were receiving csDMARDs and their counterparts in the control cohort. We hypothesized that csDMARDs exerted a protective effect against vascular types of dementia. This finding is consistent with that of a previous study by the Clinical Practice Research Datalink in the United Kingdom, which reported that methotrexate can reduce the risk of dementia among patients with RA [29]. Another study also reported that a longer use of methotrexate can reduce the risk of dementia among patients with RA [30]. We surmise that the anti-inflammatory activity of these medications is responsible for reducing the risk of dementia among patients with axSpA. Although the mechanism underlying the action of csDMARDs remains ill-defined, an overlapping unique mechanism has been hypothesized. A folate-dependent process and the generation of reactive oxygen species; inhibition of methyl-donor production; alteration of cytokines; and downregulation of matrix metalloproteinases, eicosanoids, and adhesion have been implicated in the mechanism of action of methotrexate [31]. The anti-inflammatory mechanism of methotrexate is involved in the inhibition of inflammatory cell proliferation, interference with T cell activity and cytokine secretion, augmented release of adenosine and macrophages, and inhibition of polymorphonuclear leukocyte generation [32]. A previous study reported that sulfasalazine can influence the functions of inflammatory cells, the production of cytokines and antibodies, the inhibition of synovial neovascularization, and the behavior of folate-dependent enzymes [33]. These csDMARDs were found to be involved in adenosine signal transmission and interrupted Ig synthesis to inhibit B cell activity, affect T cell activity through CD8 modulation, interfere with IL-2 production, and inhibit prostaglandin activity [32,33,34]. DMARDs and immune-modulation agents afford a protective effect against AD and all types of dementia; however, our study noted a different outcome among patients with axSpA [35]. Further research is warranted to specify the mechanism underlying the protective effects of DMARDs.

Glucocorticoids are often administered to patients with axSpA who present with uveitis or acute inflammation. Our study demonstrated that patients who receive glucocorticoids have an increased risk of developing dementia. Glucocorticoids can influence and regulate neural synaptic plasticity in the hippocampus [36]. Moreover, a previous study reported that patients receiving long-term corticosteroid treatment had a smaller hippocampal volume [37]. Because the hippocampus is a major area concerned with memory and learning, the negative effect of glucocorticoids use on the hippocampus may be one of the possible reasons behind the higher risk of dementia among these patients. 

This study has some limitations. First, data regarding the patients’ lifestyle, such as smoking habits, alcohol consumption, body mass index, and family history of dementia, are not available in NHIRD; all these factors can influence the risk of dementia. Furthermore, depression is one of the risk factors of dementia, but was not taken as a confounder in this study. Second, patients with SpA and dementia were identified using diagnostic codes, which were input by clinicians into NHIRD. Clinical information about the severity of disease, predominant syndromes, mental status, duration and stage of dementia, and neuroimaging profiles were not available. However, the certification was issued according to the standard diagnostic criteria after peer review by rheumatologists and neurologists. Third, which of these patients were using csDMARDs or other biological therapies such as anti-TNF-α treatment was not analyzed in detail. In clinical practice, it is that more than two kinds of medication are used for axSpA patients. Furthermore, patients using medications such as leflunomide, hydroxychloroquine, or azathioprine, which are not usually used for the treatment of axSpA, were still enrolled in the csDMARDs analysis. Moreover, NSAIDs, which are usually used among axSpA patients, were not analyzed in this study due to the complexity of data collection from the database. Although this study did not analyze specific medication, our database study focused the risk of dementia when using csDMARDs medications by comparing it to those not using it. Further investigation regarding specific medication for risk of dementia among axSpA patients is needed in the future. Fourth, there were RA and SLE patients coexisting in the axSpA cohort, which could be explained by the initial diagnostic errors of RA or SLE codes that were later corrected by clinical physicians as axSpA. Finally, because dementia is a slow, progressive neurodegenerative disorder, the maximum follow-up period of 7 years in our study could have failed to detect the gradual development of dementia in some patients with axSpA. This could have resulted in the risk of dementia among these patients being underestimated. To remedy this shortcoming, a cohort study with a longer follow-up period may provide more robust results.

## 5. Conclusions

This is the first study to provide findings based on real-world data on the association between csDMARDs and the risk of dementia among patients with axSpA. The use of csDMARDs can reduce the risk of development of vascular dementia in these patients. In contrast with csDMARDs, axSpA patients using glucocorticoids are still at risk of dementia. Further prospective cohort studies for specific DMARD medication and risk of dementia are recommended among axSpA patients in the future. 

## Figures and Tables

**Table 1:** Baseline demographic characteristics and comorbidities of age- and sex-matched patients in the axSpA and non-axSpA cohorts (*n* = 14,046).

Baseline Variable	Patients with axSpA (*n* = 2341)		Patients without axSpA (*n* = 11,705)		*p* Value
	No.	(%)	No.	(%)	
**Characteristics**					
Age (years)					1.00
18–30	632	27.0	3160	27.0	
31–40	563	24.0	2815	24.0	
41–50	483	20.6	2415	20.6	
51–60	322	13.8	1610	13.8	
61–70	195	8.3	975	8.3	
>70	146	6.2	730	6.2	
Sex					1.00
Male	1535	65.6	7675	65.6	
Female	806	34.4	4030	34.4	
Urbanization					<0.001
Level 1	675	28.8	3723	31.8	
Level 2	665	28.4	3297	28.2	
Level 3	370	15.8	1973	16.9	
Level 4	365	15.6	1478	12.6	
Level 5	266	11.4	1234	10.5	
Monthly income					<0.001
0 (Financially dependent)	363	15.5	2496	21.3	
1–20,000 TWD	511	21.8	4840	41.3	
20,001–40,000 TWD	1005	42.9	2476	21.2	
≥40,001 TWD	462	19.7	1893	16.2	
Comorbid medical disorders					
DM					0.440
Yes	185	7.9	871	7.4	
No	2156	92.1	10,834	92.6	
Hypertension					0.045
Yes	425	18.2	1927	16.5	
No	1916	81.8	9778	83.5	
Coronary heart disease					<0.001
Yes	191	8.2	722	6.2	
No	2150	91.8	10,983	93.8	
Parkinson’s disease					1.00
Yes	9	0.4	44	0.4	
No	2332	99.6	11,661	99.6	
Hyperlipidemia					0.001
Yes	301	12.9	1235	10.6	
No	2040	87.1	10,470	89.4	
Stroke					
Yes	91	3.9	406	3.5	
No	2250	96.1	11,299	96.5	
Autoimmune disease (RA, SLE)					<0.001
Yes	97	4.1	90	0.8	
No	2244	95.9	11,615	99.2	
Thyroid disease					<0.001
Yes	150	6.4	457	3.9	
No	2191	93.6	11,248	96.1	
Gout					<0.001
Yes	319	13.6	1242	10.6	
No	2022	86.4	10,463	89.4	
COPD					<0.001
Yes	507	21.7	2009	17.2	
No	1834	78.3	9696	82.8	
Medication therapy					
csDMARDs					<0.001
Yes	1026	43.8	83	0.7	
No	1315	56.2	11,622	99.3	
PREDNISOLONE					<0.001
Yes	1031	44.0	3451	29.5	
No	1310	56.0	8254	70.5	
METHYLPREDNISOLONE					<0.001
Yes	264	11.3	890	7.6	
No	2077	88.7	10,815	92.4	

AxSpA, axial spondyloarthritis; DM, diabetes mellitus; RA, rheumatoid arthritis; SLE, systemic lupus erythematosus.

**Table 2:** Incidence and hazard ratio for dementia between patients with and without axSpA during the 7-year follow-up (*n* = 14,046).

Presence of Dementia	Patients without axSpA	Patients with axSpA
Follow-up period		
Yes/Total	157/11,705	40/2341
Person-years	67,341	11,961
Incidence per 100,000 person-years	233	334
Crude hazard ratio (95% CI)	1.00	1.53 *** (1.08–2.17)
^a^ Model 1 Adjusted hazard ratio (95% CI)	1.00	1.73 ** (1.22–2.46)
^b^ Model 2 Adjusted hazard ratio (95% CI)	1.00	1.55 * (1.08–2.23)
^c^ Model 3 Adjusted hazard ratio (95% CI)	1.00	1.95 ** (1.31–2.91)

Notes: ^a^ Model 1 adjustments were made for propensity scores of characteristics, specifically patient age, sex, urbanization, and monthly income. ^b^ Model 2 adjustments were made for propensity scores of characteristics and comorbid medical disorders, specifically patient age, sex, urbanization, monthly income, DM, hypertension, coronary heart disease, Parkinson’s disease, hyperlipidemia, stroke, autoimmune disease (RA and SLE), COPD, thyroid disease, and gout. ^c^ Model 3 adjustments were made for propensity scores of characteristics, comorbid medical disorders, and medication therapy, specifically patient age, sex, urbanization, monthly income, DM, hypertension, coronary heart disease, Parkinson’s disease, hyperlipidemia, stroke, autoimmune disease (RA and SLE), COPD, thyroid disease, gout, csDMARD use, prednisolone use, and methylprednisolone use. * *p* < 0.05, ** *p* < 0.01, *** *p* < 0.001.

**Table 3:** Crude and adjusted hazard ratios (HRs) and 95% confidence intervals (CIs) for dementia during the 7-year follow-up.

Presence of Overall Dementia	Patients without axSpA	Patients with axSpA	
		Without csDMARDs	With csDMARDs
Crude HR (95% CI)	1.00	1.93 ** (1.29–2.89)	1.03 (0.57–1.85)
Adjusted HR (95% CI)	1.00	1.98 ** (1.32–2.99)	1.01 (0.55–1.85)
Presence of AD			
Crude HR (95% CI)	1.00	1.19 (0.27–5.17)	0.74 (0.10–5.60)
Adjusted HR (95% CI)	1.00	1.23 (0.28–5.39)	0.77 (0.10–5.89)
Presence of vascular dementia			
Crude HR (95% CI)	1.00	2.03 ** (1.33–3.09)	1.06 (0.57–1.97)
Adjusted HR (95% CI)	1.00	2.09 ** (1.36–3.20)	1.05 (0.56–1.97)

Adjustments were made for propensity scores of characteristics and comorbid medical disorders, specifically patient age, sex, urbanization, monthly income, DM, hypertension, coronary heart disease, Parkinson’s disease, hyperlipidemia, stroke, autoimmune disease (RA and SLE), COPD, thyroid disease, and gout. ** *p* < 0.01.

**Table 4:** Hazard ratios (HRs) and 95% confidence intervals (CIs) for dementia in patients with axSpA during 7-year follow-up (*n* = 2341).

Presence of Overall Dementia Medication Therapy	Hazard Ratio	(95% CI)	*p*-Value
csDMARDs	0.45	0.22–0.89	0.023
Prednisolone	2.53	1.29–4.96	0.007
Methylprednisolone	2.40	1.15–4.97	0.019
	Adjusted hazard ratio		
csDMARDs	0.45	0.22–0.90	0.024
Prednisolone	2.53	1.29–4.99	0.006
Methylprednisolone	2.31	1.11–4.80	0.025

Adjustments were made for propensity scores of characteristics and comorbid medical disorders, specifically patient age, sex, urbanization, monthly income, DM, hypertension, coronary heart disease, Parkinson’s disease, hyperlipidemia, stroke, autoimmune disease (RA and SLE), COPD, thyroid disease, and gout.

## Data Availability

Data are available upon request due to restrictions, e.g., privacy or ethical.
